# The Spirit of Science

**Published:** 1911-05

**Authors:** 


### The Spirit of Science.

In these days the practical and material gains which are brought to the community and to the individual by the advances of science are so obviously and so constantly presented to our minds that to the casual observer the promise of such material rewards might perhaps appear to be the leading motive for man's endeavor to fathom the mysteries of nature.

Yet, if we study the history of science, this is hardly the impression which we receive. Not only have most of the great pioneers followed their light in a large measure regardless of any consideration of personal gains of a material kind, but quite on the contrary, we need only turn to the records of medieval history, to find the martyr of science, driven by the spirit within him to seek the truth at all costs, and to proclaim it though his reward be torture and death at the hands of the inquisitor.

No; the prime, the fundamental motive which has impelled men to decipher the hidden meanings of nature has not been the prospect of material gain, else had they not been willing to suffer and die for the truth, but the sublime pleasure, the exalted ecstasy that comes with revelation of truth to the master mind.

The glorious transport of the hero—"das Heldenglück" as Ostwald calls it—few perhaps are privileged to experience. Yet the impulse to fathom the problems of our existence is so primitive, so fundamental a trait of the human mind, that probably none of us are quite free from it. In some of its phases the instinct of inquiry is most plainly evidenced in childhood, and is, alas! too often choked almost out of existence by the cares and petty annoyances of every-day life as we grow up. Enough, however, is left to most of us adults of the "child wonder" to enable us to complete by introspection our analysis of the elemental emotions through which the pursuit of science holds men in fascination.

First, then, and most primitive is our innate capacity for being stirred to wonder and awe by the contemplation of the grandeur of nature. Who has not experienced a feeling of mental exaltation when gazing up into the unfathomable depths of the "starry cluster" on some gloriously illumined summer night? At such moments there is an attempt on the part of the imagination to fill in the gaps where the discernment of our senses fails, and to span the untold distances over which our limited powers are incompetent to carry us. This exercise of the imagination is for some reason felt as a pleasurable experience.

But the feeling of wonder is only a first and very rude step in the journey which the individual and society have traveled to reach the vantage ground of modern scientific attainment. If there is a certain pleasure associated with a mere feeling of unsatisfied wonder, a new zest is added to the situation if through one circumstance or another a corner of the veil is lifted, and a gleam of understanding lights up our mental sight to a comprehension of the phenomenon that at first merely awed us. In one form this is pre-eminently the pleasure of the younger student of science, whose personal knowledge can as yet be readily extended by the comparatively easy means of imparting to him the knowledge gained by others.

As the student matures, he may pass more or less imperceptibly into the next stage of development, and taste of the pleasure which comes of the discovery of new truths by his own effort. The time when he passes into this phase can not be precisely defined. It will vary a great deal with the individual. Some, even with training specially directed toward the development of faculty and skill in this direction, will hardly do more than at the best add more or less material to our "tables of natural constants." In others the spirit of research is deeply ingrained. They will make truly important contributions to the treasure house of science. Their earliest efforts may perhaps merely lead them to rediscover independently what others had found before them. But as they grow in years and knowledge, they sooner or later give to us those gems in the crown of science, whose luster casts its rays through space and time, lending international renown and immortal fame to the pioneer genius. Such a man tastes not only of the joy of having himself gained knowledge by his own efforts, but he knows also the deeper satisfaction which springs from the consciousness of services rendered and benefits bestowed upon the world at large.

There is still another phase in scientific progress, whether we think of the individual or of the community, which brings its own pleasure: The sense of mastery over nature that follows upon successful application of scientific knowledge to the furtherance of human welfare. A James Watt teaches us to wield a new weapon in our aggressive warfare for the conquest of the world by man. A Pasteur gives into our hand the means of defense to ward off the last remaining enemies of mankind—foes which have baffled us so long, not through their strength or overpowering dimensions, but through their very elusive minuteness. Who shall say which is our greater debt, the one we owe to James Watt, or that due to Pasteur, and which the greater joy—that of the engineer adding to our means of gaining wealth, or of the devoted physician and pioneer of medical science, ministering to the health of nations?

Many are the fascinations of science, even to him who is content to look on while others toil and worship at her shrine. Greater, and indeed overwhelming, must be her hold on him who, with the hand of the expert, and the mind of the genius, explores new regions, and brings back from his excursions to his fellowmen at home the trophies of new truths wrested from nature's cypher record.—*Scientific American,* Editorial March 25, 1911.

---

"Ye pigge he is a pretty fowl
And wondrous goode to eate,
Hys ham is goode—lykewise hys jowl
And eke hys little feete.

But if ye trye a thousand yeare
I trow ye styll will fayle
To make a sylke purse of hys eare,
Or whissle of hys tayle."

ANONYMOUS.

